# Fear of childbirth in Iran: A systematic review of psychological intervention research

**DOI:** 10.18502/ijrm.v19i5.9250

**Published:** 2021-06-23

**Authors:** Marzieh Azizi, Mahsa Kamali, Forouzan Elyasi, Mahboobeh Shirzad

**Affiliations:** ^1^Department of Midwifery and Reproductive Health, School of Nursing and Midwifery, Tehran University of Medical Sciences, Tehran, Iran.; ^2^Boali-Sina Hospital, Mazandaran University of Medical Sciences, Sari, Iran.; ^3^Psychiatry and Behavioral Sciences Research Center, Addiction Institute, Mazandaran University of Medical Sciences, Sari, Iran.; ^4^Department of Psychiatry, School of Medicine, Mazandaran University of Medical Sciences, Sari, Iran.; ^5^Department of lnternal Medicine, School of Medicine, Mazandaran University of Medical Sciences, Sari, Iran.

**Keywords:** Fear, Childbirth, Pregnancy, Psychological intervention, Iran.

## Abstract

**Background:**

Due to the fear of childbirth (FOC) and failure to provide painless delivery in Iran, the prevalence rate of elective cesarean section (C-section) performed on request by pregnant women is on the rise. However, no systematic review assessing the results of studies in this respect has been thus far developed.

**Objective:**

To systematically review published psychological intervention research reflecting on FOC in Iran.

**Materials and Methods:**

In this systematic review, the databases of PubMed, MEDLINE, PsycINFO, Wiley, ISI Web of Science, Scopus, Science Direct, Cochrane Library, Google Scholar, and Scientific Information Database were searched to retrieve the relevant studies. Manual searches were performed to find the relevant articles and finally 21 intervention studies were reviewed.

**Results:**

Based on the modified Jadad Scale, a methodological quality (risk of bias) assessment tool, 14 and 7 studies had acceptable or good and low quality, respectively. The included articles covered fear, fear of childbirth, pregnancy, and psychological intervention in Iran. Cognitive behavioral therapy, relaxation techniques, psychological counseling, childbirth preparation classes (CPCs), mindfulness programs, and psychoeducation had been also practiced as the main types of psychological interventions for reducing FOC in pregnant women.

**Conclusion:**

There was no clear evidence to establish the most effective method for minimizing levels of FOC in pregnant women. Based on the assessment tool and since most of the studies had moderate or low quality, conducting standard and high-quality randomized controlled trials focusing on FOC in pregnant women is of most importance in Iranian population.

## 1. Introduction

Fear of childbirth (FOC) is still a critical problem during pregnancy for some women, especially the primiparous women (1–3). Based on the related literature, the prevalence rates of FOC may be different across cultures and in different countries (4–6). According to the results of Swedish studies, the prevalence of FOC in pregnant women had been reported to be about 20% and nearly 6–10% of women had intense fear (7–9); however, in Australian studies, up to 26% of women had suffered FOC (10, 11).

Investigating the causes of FOC have further indicated that factors such as young maternal age, low levels of education (3, 7), nulliparity, fear of having childbirth defects, prior negative experiences in particular prenatal complications (3, 4, 12), pre-existing psychological problems like lack of self-confidence regarding their ability for childbirth, low social support (2, 12), and a history of anxiety or depression could be associated with FOC in pregnant women (2, 13–15). FOC can correspondingly have negative effects on maternal health such as delayed obstetric–therapeutic interventions due to the unfavorable communication with medical staff resulting in prolonged labor (6, 13), higher risks of emergency cesarean section (C-section) and increased probability of instrumental vaginal delivery (3, 7, 16). The FOC also negatively influences mother–child relationships as well as child health status (4, 16).

Studies in this respect have established that women affected with FOC mostly receive treatments such as supportive counseling visits by midwives, psychologists, or psychiatrists during pregnancy (7, 17, 18); for example, the results of an investigation in Sweden, assessing the effect of counseling on FOC, had revealed that although pregnant women in counseling groups had been satisfied with treatments, this intervention had failed to statistically reduce the levels of FOC (19). Accordingly, different intervention studies have been conducted on pregnant women with FOC all over the world with the aim of minimizing the levels of this critical condition (9, 20–22).

Due to FOC and lack of painless delivery in Iran, the prevalence rate of elective C-section performed on request by pregnant women is on the rise, and this issue is in contrast with the current policy adopted by the Ministry of Health and Medical Education on C-section without indications. Moreover, most studies have preferred psychological interventions but not pharmacological or complementary medicine for FOC treatment in pregnant women since pharmacological strategies needed long-term periods for effectiveness. Although various studies with different research designs had been conducted on FOC in pregnant women in Iran (23–27), there was no review assessing their results in a systematic manner. Thus, the present study aimed to systematically review the published psychological intervention research reflecting on FOC in Iran.

## 2. Materials and Methods 

This study was a systematic review performed based on the Preferred Reporting Items for Systematic Reviews and Meta-Analyses (PRISMA) guidelines (28, 29).

### Literature and search strategy

A comprehensive literature search was conducted in electronic databases including PubMed, MEDLINE, PsycINFO, Wiley, ISI Web of Science, Scopus, Science Direct, Cochrane Library, Google Scholar, and Scientific Information Database. The latest search was accordingly performed between January and March 2020. The search was completed separately by two researchers and then checked by both of them. The search process was mainly based on systematic searches using Persian and English keywords as follows: [“clinical trial” OR “randomized controlled trial” OR “quasi-experimental” OR “pilot randomized controlled trial” OR “non-randomized trial” OR “interventional studies”] AND [“pregnancy” OR “pregnant women” OR “fear of childbirth” OR “fear of labor pain” OR “fear of delivery” OR “fear of natural childbirth” OR “fear of parturition”] AND [“educational programs” OR “cognitive behavioral therapy” OR “counseling programs” OR “relaxation” OR “mindfulness-based cognitive therapy” OR “cognitive-behavioral group therapy” OR “childbirth preparation classes” OR “couple preparation classes” OR “midwifery counseling” OR “psychological counseling” OR “psychoeducation programs”] AND [“Iran” OR “Iranian”]. In order to identify more relevant articles, the reference lists of the included studies were also searched manually.

### Study selection

Two researchers (namely MA and FE) independently screened the titles and the abstracts of the selected studies. The full-texts were reviewed for further assessment according to the inclusion and exclusion criteria in cases which were obviously relevant to the objectives of the present study.

### Inclusion and exclusion criteria

Each type of trial such as single- or double-blind clinical studies, quasi-experimental research, randomized controlled trials (RCTs), pilot studies, single- and double-blind RCTs were included in this systematic review. Studies have also been conducted on the FOC in healthy Iranian pregnant women. In this review, the researchers aimed to assess only the articles published by Iranian authors in Persian or English languages without any time restrictions. In terms of the type of interventions, studies with any type of interventions for moderating levels of FOC among pregnant women (primary outcomes) were correspondingly included. Studies recruiting pregnant women at each gestational age were also included. However, articles reflecting on women with psychological problems and also those applying non-validated FOC assessment tools were excluded.

### Data extraction and analysis 

The full-texts of the selected studies were carefully read, and the required information was extracted and summarized in descriptive tables and then cross-checked by FE. Disagreements were further resolved via discussions among the three authors. The extracted data contained the first author's name, date of publication, type of trial, sample size in each group (i.e., intervention and control groups), primary outcomes, mean ± standard deviation (SD) of FOC in each group before and after intervention, type of intervention, duration of intervention, outcome measurement, and time of outcome measurement (Table I).

### Quality (risk of bias) assessment tool 

The research team decided to assess the methodological quality (risk of bias) of the trials through the modified Jadad Scale (30, 31). This validation tool is widely used to evaluate the quality of RCTs. It is also comprised of two sections. The first section includes three direct statements such as “description of randomization of the study with appropriate methods,” “description of the double-blind study,” and “description of withdrawals and dropouts.” For the first statement, 1 point is assigned to a study if randomization has been mentioned, and an additional point can be awarded to this statement if the method of randomization has not been mentioned. For the second statement, if the study has mentioned “blinding,” 1 point is allocated and an additional point is considered if the appropriate method of blinding has been declared in the study. For the third statement, if withdrawals or dropouts have been described in the study, 1 point is given to this statement. The overall score of the first section of the Jadad Scale ranges from 0 to 5 and a higher score indicates a high-quality study (31, 32).

The second section of the modified Jadad Scale contains three additional statements about “a clear description of inclusion and exclusion criteria,” “a description of research method used to assess adverse effects,” and “a description of statistical analysis methods.” If the three statements have been cited in studies, they can receive 1 point; otherwise, the score of zero is considered. Overall scoring of this tool for each article can range from 0 (as the lowest quality) to 8 (as the highest quality). Studies with scores of 4–8 can thus represent good to excellent (i.e., high-quality) and those with scores of 0–3 can have poor or low quality (32, 33) (Table II).

## 3. Results

### Search results

**Figure 1:**
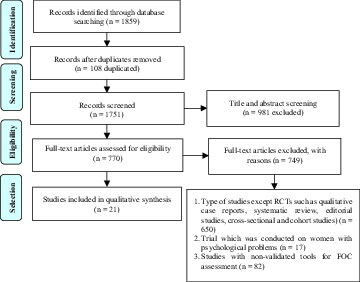
Prisma flow diagram of study search.

The systematic search resulted in 1,859 articles. After removing the duplicates (n = 108), 1,751 studies were retained. In the second stage, screening of the titles and the abstracts led to the exclusion of 981 articles. During the appraisal of the full-texts, a collection of articles was excluded if they were qualitative studies, cross-sectional and cohort studies, case reports, systematic reviews, and editorial studies (n = 650), if they had been conducted on pregnant women suffering from psychological problems (n = 17), and if they had not used validated tools (n = 82). Finally, a total number of 21 studies were included in this systematic review (Figure 1).

### Description of studies characteristics

Table I shows the results of the included studies. In this systematic review, only intervention studies aimed to reduce FOC in pregnant women as a primary outcome were assessed. They had also been published between 2008 and 2019 and included 1,782 pregnant women in intervention and control groups. The sample size also varied from 12 to 76 individuals. Based on the type of trial, 9 out of 21 included studies were quasi-experimental research (23, 34–41) while 12 were experimental or clinical trials (25, 42–52). Moreover, the blinding had been mentioned only in two studies (25, 52) but the type of blinding had not been specified in one study (52). The included studies had implemented different types of interventions, so relaxation techniques had been used as interventions for pregnant women to mitigate FOC in two studies (35, 45). In two studies, a mix of muscle relaxation techniques and guided imaginary (36) and a combination of cognitive behavioral therapy (CBT) and relaxation techniques had been employed as two interventional programs (49). In three studies using the CBT (41, 48, 50), one study had also added psychoeducation as an interventional program in frightened pregnant women (41). Other interventions included psychoeducation program (51), role-play (52), mindfulness-based cognitive-behavioral group therapy (CBGT) (34), different types of counseling programs such as individual and group psychological counseling (40, 44), self-efficacy-oriented counseling (46), and couples counseling based on problem-solving approach (23), CBGT (37), group mindfulness-based stress reduction (MBSR) (42), midwifery counseling based on solution-focused approaches (47), and reality therapy (39). In three studies, childbirth preparation classes (CPCs) had been held to reduce FOC in pregnant women (25, 38, 43).

The gestational age of pregnant women at the time of intervention had also not been reported in the inclusion criteria of three studies (37, 42, 49). In one study, each gestational age had been acceptable for being included in the study (34). The range of gestational age in pregnant women for participating in the studies based on 16 studies had been from 4 to 37 wk of gestational age.

In 20 studies, the mean ± SD of FOC before and after intervention had been correspondingly reported (23, 25, 34–37, 39–52). In one study, the mean ± SD of FOC had been reported only after the intervention and the data about the pre-intervention stage had not been mentioned (38). Except for one study whose primary outcome was pregnant women's attitudes toward FOC (38), the primary outcome in other 20 studies had been to reduce FOC. Of the included studies, the duration of implementing the interventions was variable between three and nine sessions, but most of the authors had considered an eight-session interventional protocol for their participants. Regarding the FOC measurements in the included studies, 6 articles had used the Wijma Delivery Expectancy/Experience Questionnaire (W-DEQ) (23, 41, 44, 47, 50, 51), 12 had administered Childbirth Attitude Questionnaire (CAQ) (25, 34, 38–40, 42, 43, 45, 46, 48, 49, 52), 2 had utilized a standardized FOC questionnaire (35, 37), and the Breslin's Fear Survey Schedule had been applied in one study to evaluate the FOC (36).

### Interventions for reducing FOC based on selected studies

#### CBT

A total of four studies had used CBT as an intervention program to lower the levels of fear in their participants (41, 48–50). For example, in a study, a nine-session CBT program had been accordingly considered for the intervention and control groups without any therapeutic plans. In this study, variables such as FOC, fear of labor pain, childbirth self-efficacy, and tendency to undergo C-section had been assessed. Moreover, eight individuals in the intervention group had dropped out due to the absence in two-thirds of the sessions and seven individuals had been excluded from the control group because of unwillingness to continue the study. The results of this study revealed that FOC, fear of labor pain, and tendency to have C-section had significantly decreased in the intervention group compared with the controls (p < 0.05) and also childbirth self-efficacy in the intervention group had boosted in a significant manner (p < 0.05) (50). Another study by Ghazaei et al. regarding the effectiveness of CBT and psychoeducation programs on fear of natural childbirth had been carried out on two intervention and one control groups. In this study, six individuals in the CBT group and three in the psychoeducation group had been excluded due to no attendance in two-thirds of the sessions. The results of this study also confirmed that FOC (p = 0.001) and fear of labor pain (p = 0.02) had minimized in women of the CBT group, and the tendency to have natural childbirth (p = 0.002) and childbirth self-efficacy (p = 0.001) had further increased in a significant manner compared with those in the control group. Psychoeducation as an intervention had only enhanced childbirth self-efficacy in pregnant women compared with controls. Comparing these two interventions had additionally revealed that CBT had been more effective than psychoeducation in the mentioned variables (41).

In a clinical trial, assessing the effectiveness of individual CBT on FOC in women, the results had shown that CBT had significantly reduced the levels of FOC compared with the control group (p < 0.001) (48). In another study, the effectiveness of CBT compared with relaxation techniques on delivery process in primiparous women had been investigated, and indicated no statistically significant difference between both intervention groups regarding the levels of FOC (p = 0948), however, it had been found that according to the reported mean score of FOC after intervention, CBT had been more effective than relaxation techniques in mitigating FOC in pregnant women although it had not been statistically significant (49).

#### Relaxation techniques

In three studies, the effect of relaxation on FOC among pregnant women had been evaluated (35, 36, 45). In the investigation by Khorsandi and colleagues, six sessions of relaxation classes were held as an effective strategy to cope with FOC. In these educational sessions, exercises such as deep breathing, tension-release relaxation, as well as conditional, differential, and rapid relaxation along with positive mental imagery or visualization related to delivery had been taught to the experimental group and a significant difference had been established in the mean ± SD of FOC between the two groups (p < 0.001). Also, natural delivery rate had increased considerably in the experimental group compared with the controls (p < 0.001) (45). In another study, an eight-session relaxation program had been considered for pregnant women at gestational age of 20–37 wk in the intervention group. However, the details of the educational sessions had not been specified. The mean ± SD of FOC score in the intervention group had also decreased significantly compared with those at the pre-intervention stage (p < 0.001) and also the mean score of FOC in this group had significantly declined compared with the control group (p < 0.001). Moreover, 49% and 32% of the individuals in the relaxation and control groups had respectively undergone natural delivery (p = 0.033) (35). Furthermore, one study had compared the effect of methods such as muscle relaxation and guided imagery on the FOC in primiparous women divided into three groups (two intervention and one control groups). In both experimental groups, the women had received training in two 90-min sessions for 4 wk and also practical training by educational CDs. The results had established that these techniques had been effective in reducing FOC compared with routine care (p = 0.0001) (36).

#### CPCs

A total of three studies had assessed the effect of CPCs on FOC (25, 38, 43). In Masoumi et al. study, eight educational sessions had been held for the intervention group while the control group had merely received routine care. After the intervention, five cases had been excluded due to the absence in more than one session and five individuals in the control group had refused to complete the post-intervention questionnaire. Based on the quality (risk of bias) assessment tool, this study had received the highest score in terms of quality (25). In the study by Rastegari and coworkers, the effect of CPCs on attitudes toward FOC had been assessed an hour after childbirth in women. In this study, sample selection had been correspondingly carried out using a convenience sampling method and randomization had not been cited by the research team. The intervention group had also participated in at least five sessions of CPCs during pregnancy and the control group had been chosen from pregnant women who had only received prenatal care. The results of this study had established no significant difference between the two groups based on their mean scores (p > 0.807) (38). In another study the effect of husband's presence in CPCs had been evaluated. Accordingly, eight sessions of CPCs had been held for both groups (intervention group with husband and control group without husband). The results of the study confirmed a significant decrease in the mean score of FOC in the intervention group compared with the controls (p < 0.001). Also, there was a considerable difference regarding the choice of natural delivery between the two study groups (p < 0.001) (43).

#### Psychological counseling

A variety of counseling methods had been used in five studies and their effectiveness as intervention programs in the levels of FOC among pregnant women had been assessed (23, 40, 44, 46, 47). For example, in this regard, a study had evaluated the effectiveness of couples counseling based on problem-solving approach on FOC, delivery self-efficacy, and probability of choosing natural delivery in women requesting C-section. In this study, the control group had only received routine care and the intervention group included women and their husbands participating in three weekly counseling sessions. The mean score of the couples in the intervention group had also revealed significant differences in variables such as decreased FOC, higher delivery self-efficacy, and increased probability of choosing natural delivery compared with the control group (p < 0.0001). In this investigation, three women in the control group had been removed in the follow-up stage due to preterm delivery and two in the intervention group had been excluded for pre-eclampsia and preterm labor (23). Andaroon et al. had further considered an individual counseling program for an intervention group with the aim of decreasing FOC during three sessions and routine care had also been given to the control group. In this clinical trial, the mean ± SD of FOC in the intervention group had been reported as approximately half of the score obtained by the control group, so the individual counseling program had been a significantly effective method for women affected with FOC (p < 0.001). In this study, sample dropout had been mentioned zero and all participants had been included in data analysis (44). Moreover, the effect of self-efficacy-oriented counseling to control FOC among primiparous women had been assessed in the study suggesting that six counseling sessions in the experimental group had led to a significant decrease in the mean score of FOC and increased levels of self-efficacy compared with the control group (p < 0.001) (46). A study was similarly conducted with the aim of assessing the effect of midwifery counseling based on solution-focused approaches on pregnant women. In this study, the samples had been chosen among women requesting C-section due to FOC. The women in the intervention group had received six sessions of midwifery counseling and the control group had merely received routine prenatal care. The results had also revealed significant differences between the two groups in terms of the mean scores of FOC (p < 0.001). In this study, two individuals had been excluded from the control group due to hypertension and no willingness to complete the questionnaire and three had been dropped out in the intervention group by reason of breech presentation, gestational diabetes, and absence in counseling sessions (47). In the study by Momeni, the effectiveness of group psychological counseling on anxiety and FOC had been evaluated in pregnant women. In this quasi-experimental study, the intervention group had received five sessions of psychological counseling and then Spielberger State-Trait Anxiety Inventory (STAI) and the Creative Achievement Questionnaire (CAQ) had been completed by psychological counseling in control groups. The results had shown that the score of anxiety and FOC in the intervention group had significantly reduced compared with the control group (p < 0.001). In this study, women with major psychiatric disorders had not been included. During the intervention, two individuals had also been excluded from the control group due to preterm labor (40).

#### Mindfulness program

In two studies, mindfulness had been applied as an intervention program to lower FOC among pregnant women (34, 42). In this line, a study was conducted to evaluate the effect of mindfulness-based CBT on FOC in primiparous women. Accordingly, 20 women in the intervention group had participated in eight sessions of mindfulness-based CBT and the women in the control group had only received routine prenatal care in clinics. The results had indicated the effectiveness of this intervention in reducing FOC compared with the control group (p < 0.01) (34). In the investigation by Pour-Edalati and others, the effect of MBSR on FOC in mothers with a history of natural childbirth had been explored. For this purpose, the women in the intervention group had received eight sessions of MBSR twice a week. At the end of the intervention, four individuals in the intervention group had been dropped out due to absence in more than three intervention sessions and three individuals in the control group had been excluded owing to the failure in completing the questionnaire in the post-intervention stage. The results of the study had indicated that the levels of FOC in the intervention group had been significantly lower than those in the control group (p < 0.001) (42).

#### Other interventional programs

In this respect, one study had reflected on the effect of group CBT on FOC and anxiety in pregnant women. To this end, the women in the intervention group had received eight sessions of group CBT and those in the control group had attended no counseling sessions. In this study, three individuals from the intervention group had been excluded during the intervention and three women in the control group had been removed at some point in the study due to unwillingness to complete the study. The results of this investigation had demonstrated that the group CBT had significantly and positively affected the reduction of levels of FOC (p < 0.05) and anxiety (p < 0.041) in pregnant women (37). In the study Kordi et al., childbirth psychoeducation had been used to evaluate its effectiveness on the levels of FOC in primiparous women. For this purpose, the psychoeducation program had been implemented for the intervention group in three weekly sessions while the control group had only received routine prenatal care. The results of the statistical tests had exhibited significant differences in FOC mean scores between the two groups (p = 0.001) and psychoeducation had been considered as an effective method to add to non-pharmacological therapeutic plans for pregnant women suffering from FOC. In this study, 10 individuals in the experimental group had been dropped out due to failure to attend the educational classes and 8 participants in the control group had been excluded because of abortion and displacement (51). Also, another study had revealed that the mean score of FOC in the intervention group had reduced significantly compared with the control group after reality therapy (p < 0.01) but the mean score of women who had decided to have natural childbirth after reality therapy was not significantly different from those who had continued to request elective C-section (p > 0.05). In this study, the samples had been divided into two intervention and control groups using a non-randomized purposeful sampling method. All participants had completed the study and were included in data analysis (39). In the clinical trial by Navaee et al., the effect of role-play on pregnant women had been assessed with regard to the levels of FOC and methods of delivery in pregnant women, the intervention had been carried out in three scenarios during three stages for pregnant women and the results had shown that role-play could significantly moderate FOC in the intervention group compared with the control group (p = 0.007). It had also remarkably reduced the rate of elective C-section in the intervention group (p < 0.001) (52).

### Quality (risk of bias) assessment of selected studies

A total of 21 studies were assessed using the modified Jadad Scale. Based on this methodological quality (risk of bias) tool, 14 articles had acceptable or good quality (25, 36, 39–45, 47, 48, 50–52) while the quality of 7 articles was poor (23, 34, 35, 37, 38, 46, 49). In three quasi-experimental studies, randomization had not been conducted (23, 35, 38). Although randomization had been mentioned in six studies, the method of randomization had not been stated (34, 37, 44, 46, 49, 51). Blinding had been correspondingly considered only in two studies (25, 52) and only one of them had mentioned its method of blinding (25). Although no sample dropouts had been reported in 10 studies, this issue had not been pointed out (34–36, 38, 43, 45, 46, 48, 49, 52). According to this tool guideline, if there is no sample dropout in a study, it should be noted. The inclusion and exclusion criteria had not been clearly mentioned in one study (37) and the statistical tests had been written in all studies. Since non-pharmacological interventions had been utilized in the included studies, no adverse effects had been highlighted.

**Table 1:** Characteristics of included Iranian studies


**Author, year (Ref.)**	**Type of trial**	**Sample size**	**Primary outcome**	**FOC fear before intervention (mean ± SD)**	**FOC fear after intervention (mean ± SD)**	**Type of intervention**	**Duration of intervention**	**Outcome measurement**	**Time of outcome measurement**	**Results**
**Khorsandi ** ***et al.*** **, 2008 (24)**	CT	IG: 50 CG: 50	FOC on the primiparous women	IG: 39.35 ± 6.96 CG: 40.71 ± 6.23	IG: 29.82 ± 7.18 CG: 38.03 ± 9.27	Relaxation	Nine sessions of educational program from T2	CAQ	GA of 14-28 wk (Trimester 2)	The mean score of FOC among IG was lower than CG (p < 0.001)
**Navaee ** ***et al.*** **, 2015 (52)**	Blind CT	IG1: 35 IG2: 32	Primiparous women's fear of natural delivery	IG: 35.6 ± 8.5 CG: 39.0 ± 7.0	IG: 30.6 ± 8.6 CG: 36.3 ± 8.0	Role-play education	Three sessions during seven stages	CAQ	GA of 34-36 wk	The fear score showed a higher reduction in the role-play group compared to the lecture group (p = 0.007)
**Baleghi ** ***et al.*** **, 2016 (35)**	Quasi-experimental	IG: 57 CG: 55	Reducing the FOC and increasing natural delivery	IG: 48.5 ± 13.9 CG: 58.5 ± 14.2	IG: 40.5 ± 12.4 CG: 58.7 ± 14.9	Relaxation	Eight sessions of relaxation class	FOC questionnaire	GA of 20-37 wk	The relaxation increases natural childbirth by reducing the FOC
**Rastegari ** ***et al.*** **, 2016 (38)**	Quasi-experimental	IG: 30 CG: 30	Parous women' attitude toward FOC	- IG: 34.87 ± 9.00 CG: 34.43 ± 9.31	Child birth preparation classes	Eight sessions classes	CAQ	Women after give birth	No significant difference between the two groups on attitude toward FOC (p = 0.774)
**Ghazaei, ** ***et al.*** **, 2016 (50)**	RCT	IG: 12 CG: 13	FOC and tendency toward CS in NP women	IG: 110 ± 9.59 CG: 114 ± 9.7	IG: 71 ± 17.5 CG: 107 ± 15.05	CBT	Nine sessions twice a week	W-DEQ-A	GA of 16-30 wk	FOC, tendency to CS reduced in the IG compared with the CG (p < 0.05)
**Author, year (Ref.)**	**Type of trial**	**Sample size**	**Primary outcome**	**FOC fear before intervention (mean ± SD)**	**FOC fear after intervention (mean ± SD)**	**Type of intervention**	**Duration of intervention**	**Outcome measurement**	**Time of outcome measurement**	**Results**
**Masoumi ** ***et al.*** **, 2016 (25)**	RCT	IG: 75 CG: 75	FOC	IG: 53 ± 19.3 CG: 49.1 ± 21	IG: 51.7 ± 22.4 CG: 58.7 ± 21.7	Training preparation for childbirth	Eight sessions weekly	CAQ	GA of 20 wk	The mean of fear score in IG compared to CG was significantly reduced (p = 0.007)
**Andaroon ** ***et al.*** **, 2017 (44)**	CT	IG: 45 CG: 45	FOC in primparous women	IG: 62.86 ± 12.65 CG: 63.88 ± 14.12	IG: 39.73 ± 17.08 CG: 65.66 ± 15.01	Individual counseling program	Three sessions every 2 weeks	W-DEQ-A	GA of 28-30 wk	Significant differences in FOC between two groups (p < 0.001)
**Kordi ** ***et al.*** **, 2017 (51)**	RCT	IG: 60 CG: 62	FOC intensity in primigravid women	IG: 91.04 ± 20.4 CG: 88.0 ± 16.1	IG: 83.5 ± 21.7 CG: 92.6 ± 18.4	Psychoeducation program	Three session per week	W-DEQ-A	GA of 14-28 wk	Significant difference between the IG and CG regarding the mean FOC score (p = 0.001)
**Gerami ** ***et al.*** **, 2017 (49)**	RCT	IG1: 25 IG2: 25 CG: 25	FOC	IG1: 56.96 IG2: 57.72 CG: 57.64	IG1: 40.66 IG2: 46.30 CG: 58.08	Cognitive-behavioral therapy with relaxation therapy	Eight sessions of CBT twice a week and six sessions relaxation	CAQ	- No statistically significant differences between the two IG in FOC but CBT was more effective
**Soltani ** ***et al.*** **, 2017 (46)**	CT	IG: 53 CG: 53	FOC in nulliparous women	IG: 40.35 ± 6.11 CG: 39.39 ± 6.31	IG: 30.77 ± 8.5 CG: 39.79 ± 7.06	Self-efficacy oriented counselling	Six weekly sessions	CAQ	GA of 26-32 wk	There was a significant decrease in the mean of FOC among groups (p < 0.001)
**Ahmadi ** ***et al.*** **, 2018 (23)**	Quasi-experimental	IG: 36 CG: 35	FOC, self-efficacy, and choice of delivery mode	IG: 87.58 ± 14.98 CG: 63.12 ± 17.08	IG: 63.12 ± 17.08 CG: 97.02 ± 9.63	Couples counseling based on the problem-solving approach	Three weekly sessions	W-DEQ-A	GA of 28-32 wk	Significant differences in the mean of FOC and self-efficacy between two groups (p < 0.001) and increased likely of normal delivery in IG (p < 0.001)
**Author, year (Ref.)**	**Type of trial**	**Sample size**	**Primary outcome**	**FOC fear before intervention (mean ± SD)**	**FOC fear after intervention (mean ± SD)**	**Type of intervention**	**Duration of intervention**	**Outcome measurement**	**Time of outcome measurement**	**Results**
**Aminolroayaee ** ***et al.*** **, 2018 (34)**	Quasi-experimental with pretest-posttest design	IG: 20 CG: 20	FOC in primparous women	IG: 22.8 ± 11.51 CG: 25.25 ± 9.32	IG: 15.25 ± 8.2 CG: 25.1 ± 9.26	Group cognitive therapy based on mindfulness	Eight educational session during two months	CAQ	In each GA	The training of mindfulness-based cognitive therapy has a lifetime effect on reducing FOC
**Bouzari ** ***et al.*** **, 2018 (37)**	Quasi-experimental with pretest-posttest design	IG: 15 CG: 15	FOC and anxiety in pregnant women	IG: 54.5 ± 7.67 CG: 54.0 ± 7.71	IG: 32.6 ± 3.75 CG: 53.6 ± 8.07	Behavioral group therapy	Eight sessions during two months	FOC questionnaire	- The intervention method had a statistically significant effect on FOC (p < 0.05)
**Pour-edalati ** ***et al.*** **, 2018 (42)**	experimental	IG: 20 CG: 21	FOC among single-child mothers	IG: 37.85 ± 5.59 CG: 33.19 ± 6.55	IG: 36.25 ± 5.40 CG: 33.00 ± 6.47	Group mindfulness-based stress reduction	Eight sessions twice a week	CAQ	- The level of fear after the intervention was significantly lower in the IG than in the CG (p < 0.001)
**Jamali ** ***et al.*** **, 2018 (43)**	RCT	IG: 76 CG: 76	FOC in the NP women	IG: 37.79 ± 6.68 CG: 36.49 ± 5.74	IG: 28.58 ± 6.47 CG: 32.82 ± 5.79	Childbirth preparation classes with the spouse	Eight- sessions classes	CAQ	GA of 20-30 wk	The decrease in mean of fear scores in the IG was significantly higher than the CG (p < 0.001)
**Author, year (Ref.)**	**Type of trial**	**Sample size**	**Primary outcome**	**FOC fear before intervention (mean ± SD)**	**FOC fear after intervention (mean ± SD)**	**Type of intervention**	**Duration of intervention**	**Outcome measurement**	**Time of outcome measurement**	**Results**
**Sharifzadeh ** ***et al.*** **, 2018 (47)**	CT	IG: 27 CG: 28	FOC	IG: 74.44 ± 12.53 CG: 73.50 ± 16.34	IG: 40.62 ± 11.11 CG: 68.78 ± 29.51	midwifery counseling based on solution-focused approaches	Six sessions weekly	W-DEQ-A	GA of 22-30 wk	FOC in IG was significantly decreased than CG (p = 0.001)
**Ghazaei ** ***et al.*** **, 2018 (41)**	Quasi-experimental	IG1: 13 IG2: 15 CG: 18	FOC and its related variables	IG1: 105.15 ± 8.08 IG2: 106.22 ± 12.24 CG: 103.57 ± 9.65	IG1: 62.95 ± 14.75 IG2: 85.64 ± 7.12 CG: 97.41 ± 10.21	Cognitive behavioral therapy Psychoeducation	IG1: nine sessions twice weekly IG2: six sessions twice weekly	W-DEQ-A	GA of 16-24 wk	CBT decreases the FOC (p = 0.001), fear of pain (p = 0.02) and increases the tendency to natural childbirth (p = 0.002) and self-efficacy of childbirth (p = 0.001) Psych education only led to the increase of self-efficacy of childbirth (p = 0.001) and a decrease of FOC (p = 0.01)
**Gerami ** ***et al.*** **, 2018 (48)**	RCT	IG: 25 CG: 25	FOC process	IG: 38.12 ± 7.20 CG: 36.84 ± 8.37	IG: 24.33 ± 2.22 CG: 38.92 ± 7.94	Individual cognitive behavioral therapy	Eight sessions weekly	CAQ	GA of 10-22 wk	Cognitive-behavioral education during pregnancy can be effective in reducing fear of delivery process
**Author, year (Ref.)**	**Type of trial**	**Sample size**	**Primary outcome**	**FOC fear before intervention (mean ± SD)**	**FOC fear after intervention (mean ± SD)**	**Type of intervention**	**Duration of intervention**	**Outcome measurement**	**Time of outcome measurement**	**Results**
**Momeni ** ***et al.*** **, 2018 (40)**	Quasi-experimental	IG: 48 CG: 46	The level of FOC	IG: 40.67 ± 1.1 CG: 39.79 ± 1.55	IG: 33.33 ± 1.23 CG: 40.50 ± 1.68	Group psychological counseling	Five sessions weekly	CAQ	Gestational age of 20-35 wk	Psychological counseling reduced FOC significantly (p < 0.001)
**Mahmoudjanlou ** ***et al.*** **, 2019 (39)**	Semi-experimental with pre-test, post-test design	IG: 20 CG: 19	The fear of labor pain	IG: 39.55 ± 5.61 CG: 41.63 ± 3.52	IG: 27.05 ± 3.94 CG: 41.32 ± 3.15	Reality therapy	Eight sessions twice a week	CAQ	GA ≥ 4 wk	The fear of labor pain in the IG was significantly different from that of the CG (p < 0.05)
**Boryri ** ***et al.*** **, 2019 (36)**	Quasi-experimental	IG1: 60 IG2: 60 CG: 60	FOC in primiparous women	IG1: 39.5 ± 6.91 IG2: 44.00 ± 9.30 CG: 41.56 ± 9.37	IG1: 30.45 ± 6.56 IG2: 34.78 ± 9.36 CG: 41.78 ± 8.69	Muscle relaxation guided Imagery	Two 90-min training sessions for four weeks	Brislin's questionnaire	GA of 26-32 wk	Mean scores of the FOC after the intervention showed a significant difference among the groups (p = 0.0001)
SD: Standard deviation, CT: Clinical trial, IG: intervention group, CG: Control group, FOC: Fear of childbirth, CAQ: Childbirth attitude questionnaire, GA: Gestational age, W-DEQ-A: Wijma delivery expectancy/experience questionnaire, CS: Cesarean section, RCT: Randomized controlled trial, NP: Nulliparous										

**Table 2:** Quality assessment of included studies by the modified Jadad Scale


**Author, year (Ref)**	**Was the study described as randomized?** **(yes:1, no: 0)**	**Was the method of randomization appropriate?** **(yes: 1, no: -1, not described: 0)**	**Was the study described as blinding?** **(yes: 1, no: 0)** **Double-blind = 1 score, single-blind = 0.5**	**Was the method of blinding appropriate?** **(yes: 1, no: -1, not described: 0)**	**Was there a description of withdrawals and drop outs?** **(yes: 1, no: 0)**	**Was there a clear description of the inclusion/exclusion criteria?** **(yes: 1, no: 0)**	**Was the method used to assess adverse effects described?** **(yes: 1, no: 0)**	**Was the approaches of statistical analysis described?** **(yes: 1, no: 0)**	**Total Score**
**Ahmadi ** ***et al.*** **, 2018 (23)**	0	0	0	0	1	1	0	1	3
**Aminolroayaee ** ***et al.*** **, 2018 (34)**	1	0	0	0	0	1	0	1	3
**Andaroon ** ***et al.*** **, 2017 (44)**	1	0	0	0	1	1	0	1	4
**Baleghi ** ***et al.*** **, 2016 (35)**	0	0	0	0	0	1	0	1	2
**Boryri ** ***et al.*** **, 2019 (36)**	1	1	0	0	0	1	0	1	4
**Bouzari ** ***et al.*** **, 2018 (37)**	1	0	0	0	1	0	0	1	3
**Pour-edalati ** ***et al.*** **, 2018 (42)**	1	1	0	0	1	1	0	1	5
**Jamali ** ***et al.*** **, 2018 (43)**	1	1	0	0	0	1	0	1	4
**Khorsandi ** ***et al.*** **, 2008 (45)**	1	1	0	0	0	1	0	1	4
**Rastegari ** ***et al.*** **, 2016 (38)**	0	0	0	0	0	1	0	1	2
**Soltani ** ***et al.*** **, 2017 (46)**	1	0	0	0	0	1	0	1	3
**Sharifzadeh ** ***et al.*** **, 2018 (47)**	1	1	0	0	1	1	0	1	5
**Ghazaei ** ***et al.*** **, 2018 (41)**	1	1	0	0	1	1	0	1	5
**Ghazaei ** ***et al.*** **, 2016 (50)**	1	1	0	0	1	1	0	1	5
**Kordi ** ***et al.*** **, 2017 (51)**	1	0	0	0	1	1	0	1	4
**Gerami ** ***et al.*** **, 2018 (48)**	1	1	0	0	0	1	0	1	4
**Gerami ** ***et al.*** **, 2017 (49)**	1	0	0	0	0	1	0	1	3
**Mahmoudjanlou ** ***et al.*** **, 2019 (39) **	1	1	0	0	1	1	0	1	5
**Masoumi ** ***et al.*** **, 2016 (25)**	1	1	1	1	1	1	0	1	7
**Momeni ** ***et al.*** **, 2018 (40)**	1	1	0	0	1	1	0	1	5
**Navaee ** ***et al.*** **, 2015 (52)**	1	1	1	0	0	1	0	1	5

## 4. Discussion

This systematic review reflected on published psychological intervention studies with an emphasis on the levels of FOC in pregnant women. Accordingly, the review of the related literature showed that different interventions had been conducted on FOC among pregnant women. Based on the literature review regarding the effect of CBT on FOC in pregnant women, most domestic and international studies had acknowledged its positive effect. For example, the effectiveness of the Internet-based CBT (ICBT) had been mentioned in three studies (53–55). According to a prospective RCT comparing the effectiveness of ICBT and counseling and standard prenatal care in pregnant women at gestational age 17–20 wk, the results had revealed that ICBT was an effective method in promoting the general health status of women with FOC which decreased the rate of C-section on maternal request for non-medical reasons (54). Another study had further reported that ICBT had led to more realistic attitudes toward natural childbirth, increased self-confidence, and ability to cope with labor process (55). In contrast with the aforementioned results, one study had revealed contradictory results in which ICBT had no significant effect on the levels of FOC in the intervention group (53). Other studies had similarly demonstrated that CBT had significantly reduced the mean score of FOC, decreased labor pain, shortened the second stage of labor, and ultimately reduced the levels of fear of blood and injection phobia (56, 57).

All Iranian studies included in this systematic review had confirmed the effectiveness of counseling in reducing FOC among pregnant women. In this regard, a longitudinal study in Sweden comparing two groups of women with and without prenatal counseling had established that women in the counseling group had reported higher FOC one year after childbirth and the elective C-section was more prevalent among the counseling group (19). Also, in another study, pregnant women receiving midwifery counseling had been satisfied with their treatment but they had reported more frightening experiences of childbirth and had shown symptoms of post-traumatic stress disorder compared with the control group (18). The results of these two studies had been in contrast with the findings of the Iranian studies included in this review.

Another intervention in the selected studies was the assessment of the effectiveness of mindfulness strategy on FOC. In this regard, the results of a study aimed to assess the effect of mindfulness-based childbirth education on self-efficacy and FOC was similar. It had demonstrated the effectiveness of this method in decreasing FOC, boosting self-efficacy, and enhancing self-control during natural childbirth (23). Moreover, another study had indicated the effectiveness of mindfulness in improving psychological health status of pregnant women for natural delivery (58).

The literature review had found that relaxation used as a method associated with psychoeducation was an effective strategy in preparing women for motherhood and decreasing their severe FOC (59, 60). In this regard, midwife-led (20) and group-based psychoeducation along with relaxation (22, 59) had been practiced in other countries, implying a positive effect on severe FOC and increased maternal adjustment to labor pain and natural delivery and also improved psychological status of pregnant women such as decreased symptoms of depression and better childbirth self-efficacy (59).

In Iran, CPCs are generally started from week 20 of gestation for pregnant women and they are free to participate in such classes. CPCs consists of eight weekly sessions about anatomical and physiological changes during pregnancy, prenatal nutrition, mental health, alarming symptoms in pregnancy, advantages and disadvantages of vaginal delivery and C-section, familiarity with various stages of natural childbirth, postpartum health, encouragements for breastfeeding, neonatal care, as well as family planning (20, 61). This systematic literature review showed that CPCs had been used for coping with labor and improving mother–child health status (62, 63). Based on a Swedish study comparing the effectiveness of CPCs and routine prenatal care, the results had revealed that the given method had decreased parental stress in the early stage of parenthood but it had achieved no effectiveness in terms of the need to use analgesia during labor (62). The results of an Iranian study using CPCs and measuring its effects on mother–child health status had further revealed the effectiveness of this method in mother–child health status and lowered rates of C-section requests by pregnant women (63).

### Clinical implication

This study can be a good resource for psychiatrists to review the current interventional studies on FOC and select the best interventions choices for the management of their patients. Also, the findings of this systematic review presented the importance of conducting high-quality interventional studies to improve the psychological status of pregnant women in Iranian population.

### Strength and limitations 

The major strength of this study was a systematic review of the published Iranian intervention research on FOC using the PRISMA guidelines. Due to differences and heterogeneities in the included studies with respect to their interventions, meta-analysis was not considered.

## 5. Conclusion

Based on this systematic review, different interventions had been used for reducing FOC among pregnant women and most of them had shown effective results in this respect. Additionally, there was no clear evidence to show the most effective method for decreasing levels of FOC among pregnant women. As most selected studies had moderate or low quality based on the quality (risk of bias) assessment tool, conducting standardized and high-quality RCTs on FOC in pregnant women in Iranian population is of utmost importance.

##  Conflict of Interest

The authors declare that there is no conflict of interest.
